# Tuberculosis screening among people who experience homelessness in Brno: a 20-year public health intervention, Czechia, 2005 to 2024

**DOI:** 10.2807/1560-7917.ES.2025.30.37.2500119

**Published:** 2025-09-18

**Authors:** Veronika Šponiar Ovesná, Renata Ciupek, Alena Holčíková, Hana Jirková

**Affiliations:** 1 Regional Public Health Authority of South Moravian Region based in Brno, Czechia; 2 Military Faculty of Medicine, University of Defence, Hradec Králové, Czechia

**Keywords:** Tuberculosis, Homelessness, Screening, Public health intervention, Czechia, Brno

## Abstract

**INTRODUCTION:**

Tuberculosis (TB) is curable and preventable, yet remains a health concern in vulnerable populations. Individuals experiencing homelessness are at increased risk owing to medical, environmental and social factors.

**AIM:**

We aimed to evaluate a 20-year TB screening programme in people experiencing homelessness in Brno, Czechia (2005–2024), and analyse additional TB diagnoses in this population made outside the project, to inform public health strategies for TB control in vulnerable groups in a low-incidence country.

**METHODS:**

Clinical examination and chest X-ray screening were offered to people without stable housing, incentivised by meal vouchers. Individuals with pathological findings underwent further diagnostic evaluation and treatment. Demographic and clinical data were collected. Additional TB diagnoses made in this population through other detection methods were analysed for comparison.

**RESULTS:**

Between 2005 and 2024, of 3,918 individuals approached, 2,664 participated in screening (average participation rate: 68.0%), and 18 were diagnosed with TB through the project. Another 132 individuals experiencing homelessness were diagnosed with TB through other pathways, yielding 150 diagnoses, representing 19.3% of TB notifications in Brno. The estimated TB incidence among people experiencing homelessness was 24.4 times higher than in the general population (95% confidence interval: 20.5–28.9). Despite a citywide decline in TB incidence, the proportion of TB diagnoses among people who experience homelessness increased over time.

**CONCLUSION:**

Homelessness is a risk factor for TB in low-incidence settings. This long-term screening initiative proved feasible and valuable, demonstrating how outreach-based screening can support early detection and contribute to TB prevention among socio-economically marginalised populations.

**Key public health message**

**What did you want to address in this study and why?**

We wanted to find out how many people who experience homelessness in Brno were affected by tuberculosis (TB) over the past 20 years, and whether a regular screening programme could help detect the disease early.

**What have we learnt from this study?**

Between 2005 and 2024, we screened 2,664 people who experience homelessness in Brno and found 18 people developing TB. We also looked at TB cases found outside the screening programme and found that people experiencing homelessness made up 19.3% of all TB cases in the city, even though they represent less than 1% of the population.

**What are the implications of your findings for public health?**

People who experience homelessness were ca 24 times more likely to develop TB than the general population. Regular TB check-ups helped to detect illness early and may have stopped it from spreading. Long-term cooperation between health and social services made it possible to reach people who often face barriers to accessing healthcare and to support them more effectively.

## Introduction

Tuberculosis (TB) is a curable and preventable infectious disease, yet it continues to rank as the second most frequent infectious cause of death worldwide, following COVID-19. According to the World Health Organization (WHO), an estimated 10.8 million people developed TB in 2023, with 1.25 million deaths attributed to the disease globally [1].

The clinical spectrum of TB includes TB infection without disease, subclinical TB and active TB disease. People with TB infection may not experience any symptoms, but some may progress to active disease, particularly if they are immunocompromised or exposed to other risk factors. The lifetime risk of developing active TB for people with the infection is estimated at 5–10% in the general population and is considerably higher among immunocompromised individuals [1,2].

Czechia, with a TB incidence of ca four individuals per 100,000 population, ranks among the countries with the lowest TB incidence in Europe [1]. Although TB incidence has generally declined across Europe over the past two decades, certain urban areas continue to experience a disproportionate burden among socially disadvantaged groups, including people experiencing homelessness [3-5].

Homelessness is a persistent socioeconomic challenge in urban centres of high-income countries and is associated with numerous risk factors for TB infection and progression to active TB disease. These include poor physical and mental health, inadequate hygiene, substance use (alcohol or drugs), social exclusion, poverty, malnutrition and limited access to medical care. Furthermore, TB transmission may be facilitated in overcrowded shelters and temporary accommodation settings [6,7].

Brno, with over 400,000 residents, is the second-largest city in Czechia [8]. It is known as a cultural, academic and technological hub, as well as a key transit point linking Prague (Czechia), Ostrava (Czechia), Vienna (Austria) and Bratislava (Slovakia). These factors create a dynamic, multicultural urban environment, also including migrants from countries with high TB incidence.

Homelessness has been a growing concern in Brno. Point-in-time counts conducted by the city reported 1,179 people who experience homelessness in 2006 (0.3% of total residents), 1,354 in 2010 (0.4%), 1,950 in 2014 (0.5%), and 1,657 in 2018 (0.4%). Owing to limitations in data collection, however, the actual number was estimated to range between 2,000 and 3,000 individuals [9]. These figures demonstrate a persistent or at times rising trend in homelessness, which reinforces the need for targeted public health interventions.

Since 2005, the City of Brno, Czechia, has implemented a TB screening programme for people experiencing homelessness, aiming to promptly identify and treat individuals with TB, thereby reducing TB transmission and contributing to public health protection. For clarity, this intervention will hereafter be referred to as the ‘Project’. The aim of this study was to evaluate the outcomes of the first 20 years of this screening programme (2005–2024) and to analyse additional TB diagnoses in this population made outside the Project, in order to inform public health strategies for TB control in vulnerable groups in a low-incidence setting.

## Methods

### Study design and setting

This study analysed data collected within a long-term public health intervention aimed at TB screening among individuals experiencing homelessness in Brno, Czechia (the Project). The Project has been implemented continuously since 2005 through a collaboration between the Regional Public Health Authority of the South Moravian Region, the Department of Social Services of the Brno City Municipality, and Pulmonary Outpatient Clinic at Poliklinika Zahradníkova in Brno. It was originally established under Project BCA 04–05/3 agreement between the Ministry of Health of the Czech Republic and the WHO Regional Office for Europe.

Screening rounds were organised twice per year (spring and autumn), and each round lasted approximately 2 months, with examinations conducted on one afternoon per week. The Project was paused in 2020 and 2021 during COVID-19-related restrictions and resumed once public health conditions allowed. The scope and logistics of the Project remained generally consistent across the study period.

According to the Project protocol, individuals who had been screened without TB diagnosis were eligible to participate in the Project again after 2 years. Duplicate entries were minimised through administrative coordination, and individuals with any history of TB were not excluded.

### Recruitment and inclusion

Individuals experiencing homelessness were invited to participate by municipal social workers and referred to a designated outpatient pulmonology clinic. To encourage participation, meal vouchers funded by the City of Brno were provided upon completion of the medical examination. Participation was voluntary, and informal oral consent was obtained on site.

The Project targeted adults meeting the European Typology of Homelessness and Housing Exclusion (ETHOS) criteria, which include individuals sleeping rough, staying in emergency shelters, institutional facilities, or other unstable housing arrangements without permanent accommodation [9]. We applied this definition both for inclusion in the Project and for estimating the size of the population of people experiencing homelessness in Brno.

### Screening procedures and diagnostics

All participants underwent a structured medical interview and clinical examination, followed by a chest X-ray. Screenings were conducted outside of standard clinic hours to minimise disruption to routine healthcare services. Interviews collected basic identification data and demographic information (sex, age), along with questions about TB-related symptoms, which include cough, fever, night sweats, dyspnoea, chest pain, haemoptysis and weight loss, consistent with standard clinical criteria.

Participants with outside the normal range of clinical or radiological findings were hospitalised for further diagnostic evaluation at the Department of Pulmonary Diseases and Tuberculosis, University Hospital Brno. This included comprehensive clinical assessment and microbiological testing. Sputum samples were obtained for smear microscopy and culture (if spontaneous sputum was not available, induced sputum or bronchoalveolar lavage was performed as clinically indicated). Additional diagnostic methods included PCR testing for *Mycobacterium tuberculosis* and, when appropriate, histopathological examination (e.g. from pleural fluid or lymph node biopsy). Cases with negative cultures but clinical and radiographic evidence consistent with TB were classified based on clinical judgement, in line with national and WHO guidelines [10,11].

All individuals diagnosed with TB were hospitalised for isolation and started on standard treatment, as mandated by national TB control regulations [12,13]. Medical care, including diagnostics, hospitalisation and TB treatment, was fully covered by public health insurance.

Participants with abnormal chest X-ray findings but without bacteriological confirmation of TB were referred for a comprehensive diagnostic evaluation. In several cases, radiological changes were attributable to other pulmonary conditions, such as lung cancer or non-specific chronic lung damage, and appropriate treatment was initiated. If no disease requiring therapy was identified and TB was excluded, individuals were not treated but remained under medical supervision as clinically indicated. According to project protocol, they were allowed to re-enter the screening programme after 2 years.

### Case definition

Active TB was classified according to Czech national legislation and aligned with European standards [10,11]. A confirmed TB case was defined as a person with *M. tuberculosis* complex identified by culture, smear microscopy or PCR from respiratory or other biological specimens. Probable cases were those without bacteriological confirmation but with clinical, radiological or histological findings suggestive of active TB, as assessed by a specialist physician. Both confirmed and probable TB diagnoses were included in the analysis.

Tuberculosis was further classified by site of disease using ICD-10 diagnostic codes – pulmonary TB (A15.0–A16.9), including tracheobronchial, laryngeal and pleural TB; and extrapulmonary TB (A17–A19), including TB of lymph nodes, meninges, genitourinary system, bones and joints, digestive system and miliary TB without pulmonary involvement.

We included only new (incident) TB episodes in the analysis; no recurrences were recorded as separate cases during the study period.

### Additional individuals diagnosed with tuberculosis

To contextualise the Project outcomes, we reviewed data on additional individuals experiencing homelessness who were diagnosed with TB in Brno between 2005 and 2024 through other detection methods. These included passive detection (individuals presenting with symptoms), screening upon prison admission, autopsy and other surveillance mechanisms. Homeless status was identified through information provided in mandatory TB notification reports to the Regional Public Health Authority.

### Epidemiological follow-up

All individuals diagnosed with TB (whether identified through the Project or through other detection routes) were reported to the Regional Public Health Authority, as required by national public health legislation [12,13]. Notifications were submitted by the diagnosing physician and laboratory. Standard public health procedures followed, including contact tracing, care coordination, and long-term follow-up by regional epidemiologists. Each person diagnosed with TB was monitored until treatment completion, death or loss to follow-up, typically a 12–48-month period, depending on the clinical course and treatment response, and included coordination between epidemiologists, clinical facilities and social service providers. Public health measures were implemented as needed.

### Data collection

For each screening round of the Project, we recorded data on the number of invited individuals, participation rates and clinical or radiological findings. Basic demographic data (sex, age, nationality) were obtained during the initial medical examination. The administration, coordination and evaluation of the Project were overseen by epidemiologists from the Regional Public Health Authority of the South Moravian Region.

For all individuals diagnosed with TB, regardless of detection method, the diagnosing physician completed a mandatory reporting form and submitted it to the Regional Public Health Authority. These reports contained comprehensive information including identification, age, sex, nationality, housing history, clinical presentation, risk behaviours (e.g. alcohol or drug use, smoking), vaccination status and detailed diagnostic findings (e.g. radiographic, bacteriological, molecular). The form also documented the route of detection (e.g. symptomatic presentation, prison screening, autopsy), details of treatment (including hospitalisation, adverse reactions, treatment duration) and final treatment outcome. This information was entered into the national TB register and cross-verified by epidemiologists using clinical documentation, discharge summaries and death certificates.

Population estimates for the general Brno population were obtained from the Czech Statistical Office [8].

### Statistical analysis

All data were anonymised before analysis. For the Project, we used descriptive statistics to summarise demographic characteristics, participation rates and TB detection outcomes. TB prevalence was calculated annually as the number of individuals diagnosed with TB per 1,000 people screened.

To estimate TB incidence among people experiencing homelessness, we used the number of individuals diagnosed with TB (identified via the Project or other methods) as the numerator, and the estimated size of the homeless population as the denominator. This population was assumed to range from 2,000 to 3,000 individuals, based on point-in-time municipal surveys conducted in 2006, 2010, 2014 and 2018, and expert estimates from municipal social service providers [9]. TB incidence for the general Brno population was calculated using official surveillance data from national TB register and population estimates from the Czech Statistical Office [8].

The relative risk of TB among people experiencing homelessness compared with the general Brno population was calculated as the ratio of incidence rates. We report the average relative risk for the entire 20-year period (2005–2024). We calculated 95% confidence intervals (CI) using the Mid-P exact test in OpenEpi (version 3.01). All other calculations were performed in Microsoft Excel.

## Results

### Participation in the project

Between 2005 and 2024, a total of 3,918 individuals experiencing homelessness were invited to participate in the Project. Of these, 2,664 individuals were screened through clinical examination and chest X-ray, resulting in an overall participation rate of 68.0%. The participation rate varied by year, ranging from 42.3% in 2015 to 79.2% in 2010.

The majority of participants were men (n = 2,113; 79.3%), whereas women accounted for 551 (20.7%). The most represented age groups were 45–54 years (n = 804; 30.2%) and 55–64 years (n = 681; 25.6%). These demographic patterns are consistent with the overall profile of the population of people who experience homelessness in Brno. No screening was conducted in 2020 or 2021 because of restrictions related to the COVID-19 pandemic. Detailed demographic characteristics of participants in the Project over years are provided in Table 1.

**Table 1:** Characteristics of participants in the Project, Brno, 2005–2024 (n = 2,664)

Year	Number invited	Number screened	Participation rate (%)	Sex of participants		Age of participants (years)					
				Male	Female	15–24	25–34	35–44	45–54	55–64	≥ 65
2005	744	566	76.1	433	133	40	78	146	159	114	29
2006	290	173	59.7	150	23	22	35	39	53	20	4
2007	349	247	70.8	187	60	20	28	42	74	63	20
2008	199	141	70.9	103	38	6	18	25	39	41	12
2009	285	199	69.8	148	51	9	17	38	58	60	17
2010	250	198	79.2	165	33	9	16	51	65	48	9
2011	200	154	77.0	128	26	5	18	19	55	49	8
2012	308	147	47.7	125	22	1	20	31	53	35	7
2013	259	192	74.1	166	26	9	16	43	61	57	6
2014	138	103	74.6	80	23	2	17	20	33	25	6
2015	201	85	42.3	73	12	3	12	13	27	30	0
2016	113	81	71.7	63	18	2	6	19	21	31	2
2017	150	112	74.7	92	20	2	12	25	35	32	6
2018	102	73	71.6	63	10	1	9	18	18	21	6
2019	113	78	69.0	58	20	1	5	19	20	26	7
2020	NA										
2021	NA										
2022	47	27	57.4	21	6	0	0	8	5	8	6
2023	57	29	50.9	19	10	0	1	5	9	8	6
2024	113	59	52.2	39	20	0	6	15	19	13	6
**Total**	**3,918**	**2,664**	**68.0**	**2,113** **(79.3%)**	**551** **(20.7%)**	**132** **(5.0%)**	**314** **(11.8%)**	**576** **(21.6%)**	**804** **(30.2%)**	**681** **(25.6%)**	**157** **(5.9%)**

NA: not applicable.

NA indicates years when the project was not conducted because of COVID-19 pandemic-related restrictions (2020–2021).

### People diagnosed with tuberculosis through the project

Between 2005 and 2024, a total of 18 individuals were diagnosed with TB through the Project, corresponding to a yield of 6.8 per 1,000 persons screened. The highest number of diagnoses (n = 4) occurred in the first year (2005), followed by years with one or two diagnoses and several years with none. Annual TB prevalence among participants varied substantially over time. The highest yields were observed in 2018 (27.4 per 1,000 screened) and 2019 (25.6 per 1,000 screened), while there were several years when no participants were diagnosed, particularly after 2019.

All 18 individuals diagnosed through the Project were Czech citizens, and 17 of them were men. Most were aged 55–64 years (10 persons; 14.7 per 1,000 screened), followed by 45–54 years (five persons; 6.2 per 1,000) and 35–44 years (three persons; 5.2 per 1,000). The mean age at diagnosis was 53.8 years (range: 37–63). Detailed breakdowns by year, sex and age group are presented in Table 2.

**Table 2:** Descriptive statistics of individuals diagnosed with tuberculosis through the Project, Brno, 2005–2024 (n = 18)

Year	Number diagnosed	Prevalence (per 1,000 screened)	Sex of cases		Age of cases (years)					
			Men	Women	15–24	25–34	35–44	45–54	55–65	≥ 65
2005	4	7.1	3	1	0	0	1	1	2	0
2006	1	5.8	1	0	0	0	1	0	0	0
2007	1	4.0	1	0	0	0	0	0	1	0
2008	0	0	0	0	0	0	0	0	0	0
2009	2	10.1	2	0	0	0	0	0	2	0
2010	1	5.1	1	0	0	0	0	1	0	0
2011	1	6.5	1	0	0	0	0	1	0	0
2012	2	13.6	2	0	0	0	0	1	1	0
2013	1	5.2	1	0	0	0	0	0	1	0
2014	0	0	0	0	0	0	0	0	0	0
2015	0	0	0	0	0	0	0	0	0	0
2016	1	12.3	1	0	0	0	1	0	0	0
2017	0	0	0	0	0	0	0	0	0	0
2018	2	27.4	2	0	0	0	0	1	1	0
2019	2	25.6	2	0	0	0	0	0	2	0
2020	NA									
2021	NA									
2022	0	0	0	0	0	0	0	0	0	0
2023	0	0	0	0	0	0	0	0	0	0
2024	0	0	0	0	0	0	0	0	0	0
**Total**	**18**	**6.8**	**17**	**1**	**0**	**0**	**3**	**5**	**10**	**0**

NA: not applicable.

NA indicates years when the project was not conducted because of COVID-19 pandemic-related restrictions (2020–2021).

All individuals diagnosed with TB through the Project reported symptoms consistent with TB but had not previously sought medical attention. The most common symptoms were cough (n = 14), shortness of breath (n = 8), fever or night sweats (n = 8), chest pain (n = 2), haemoptysis (n = 2) and weight loss (n = 2).

Seventeen individuals were diagnosed with pulmonary TB disease (nine confirmed by smear microscopy, seven by culture only, and one based on clinical and radiological findings despite negative microbiology). One person was diagnosed with tuberculous pleuritis.

Reported risk factors included current smoking (n = 18) and alcohol use (n = 15). Importantly, no one reported drug use. Ten individuals had a history of TB vaccination.

Long-term follow-up data show that 13 of the 18 individuals successfully completed treatment and recovered. In two instances, treatment remained incomplete because the patients absconded from care and could not be traced. Three people died: two deaths were directly attributed to TB, and one was due to another cause.

### Additional individuals experiencing homelessness diagnosed with tuberculosis

**Figure 1:**
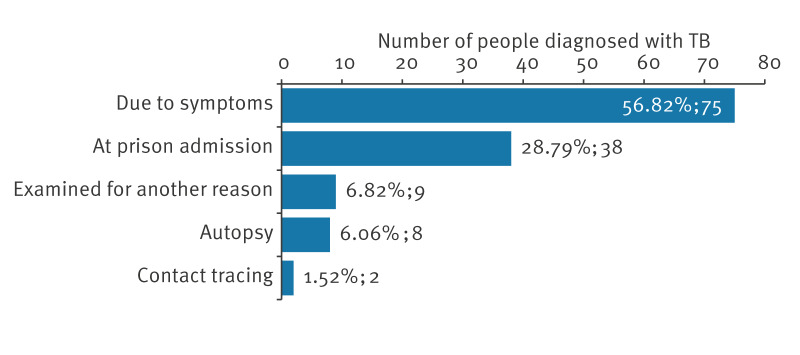
Method of tuberculosis detection among additional individuals experiencing homelessness, Brno, 2005–2024 (n = 132)

In addition to the individuals diagnosed with TB through the Project, another 132 individuals experiencing homelessness were diagnosed with TB in Brno between 2005 and 2024. Of these, 75 (56.8%) were diagnosed through passive case detection (i.e. individuals presented for medical care due to symptoms). The remaining diagnoses were made during prison entry screening (n = 38), autopsy (n = 8), contact tracing (n = 2) or other healthcare encounters (n = 9), as illustrated in Figure 1.

TB: tuberculosis.

Among these 132 individuals, 117 (88.6%) were men. The most common age group at the time of diagnosis was 45–54 years (n = 50), followed by 55–64 years (n = 35) and 35–44 years (n = 22). The mean age at diagnosis was 50.3 years (range: 20–80). The year with the highest number of new diagnoses was 2022 (n = 15), while only one person was diagnosed in 2005. Detailed demographic data are shown in Table 3.

**Table 3:** Descriptive statistics of the additional individuals experiencing homelessness diagnosed with tuberculosis, Brno, 2005–2024 (n = 132)

Year	Number diagnosed	Sex of cases		Age of cases (years)					
		Men	Women	15–24	25–34	35–44	45–54	55–65	≥ 65
2005	1	1	0	0	0	0	0	0	1
2006	4	3	1	0	0	1	1	2	0
2007	14	12	2	0	1	1	6	5	1
2008	7	7	0	0	1	2	3	1	0
2009	9	9	0	1	0	2	2	3	1
2010	9	9	0	0	1	1	5	2	0
2011	7	7	0	0	1	1	4	1	0
2012	4	3	1	0	0	0	1	2	1
2013	2	2	0	0	0	0	1	1	0
2014	2	2	0	0	0	0	1	1	0
2015	10	9	1	0	1	1	2	5	1
2016	5	4	1	0	0	1	2	2	0
2017	11	10	1	0	2	2	2	5	0
2018	4	1	3	0	0	0	4	0	0
2019	4	4	0	0	0	2	0	1	1
2020	3	3	0	0	0	0	1	1	1
2021	7	6	1	0	2	0	3	0	2
2022	15	13	2	1	3	4	5	2	0
2023	10	8	2	0	0	3	5	1	1
2024	4	4	0	1	0	1	2	0	0
**Total**	**132**	**117** **(88.6%)**	**15 ** **(11.4%)**	**3 ** **(2.3%)**	**12 ** **(9.1%)**	**22 ** **(16.7%)**	**50 ** **(37.9%)**	**35 ** **(26.5%)**	**10 ** **(7.6%)**

Data on nationality were available for all individuals diagnosed with TB: 100 (75.8%) were Czech citizens. The remaining 32 individuals were foreign nationals, most commonly Ukrainian (n = 10), Slovak (n = 7) and Romanian (n = 5), as summarised in Table 4.

**Table 4:** Nationality distribution of the additional individuals experiencing homelessness diagnosed with tuberculosis, Brno, 2005–2024 (n = 132)

Nationality	Number
Czech	100
Ukrainian	10
Slovak	7
Romanian	5
Vietnamese	3
Moldovan	2
Bulgarian	1
Kenyan	1
Lithuanian	1
Polish	1
Russian	1
**Total**	**132**

The majority of these diagnoses (124 of 132) involved pulmonary TB. These were confirmed by microscopy (n = 56), culture (n = 43) or histological examination (n = 11). In 14 individuals, TB was diagnosed clinically based on radiological and symptomatic findings despite negative laboratory results. There were also two persons with TB pleuritis, one with TB of the larynx/trachea/bronchi, and one with unspecified respiratory TB. Four individuals were diagnosed with extrapulmonary TB affecting the meninges, digestive system, skin or other organs.

Frequent risk factor profiles among these individuals were tobacco use (n = 121; 91.7%) and alcohol consumption (n = 104; 78.8%), and a quarter (n = 33; 25.0%) reported drug use. In addition, 94 persons (71.2%) had received prior TB vaccination.

Long-term follow-up data show that 95 individuals (72.0%) completed treatment and recovered. Four individuals discontinued treatment prematurely and could not be located. In total, 30 individuals died: 14 due to TB and 16 from unrelated causes. Three remained in treatment at the time of reporting.

### Overall tuberculosis incidence among people experiencing homelessness in Brno

Overall, 150 individuals experiencing homelessness in Brno were diagnosed with TB between 2005 and 2024, 18 through the Project’s screening and 132 through other means. Compared with the total number of people diagnosed with TB in the city, those experiencing homelessness accounted for ca 19.3% of all reported TB diagnoses in Brno during this period. This proportion varied by year, ranging from 6.8% in 2006 to 53.6% in 2022 (Table 5).

**Table 5:** Tuberculosis diagnoses among people experiencing homelessness compared with all tuberculosis diagnoses in Brno, 2005–2024 (n = 150)

Year	Project	Other means	Total diagnosed among people who experience homelessness	Total TB diagnoses in Brno	TB incidence (per 100,000) in Brno	Proportion of TB diagnoses among people who experience homelessness (%)
2005	4	1	5	71	19.31	7.0
2006	1	4	5	73	19.90	6.8
2007	1	14	15	66	18.00	22.7
2008	0	7	7	53	14.38	13.2
2009	2	9	11	43	11.60	25.6
2010	1	9	10	44	11.85	22.7
2011	1	7	8	37	9.74	21.6
2012	2	4	6	37	9.76	16.2
2013	1	2	3	31	8.19	9.7
2014	0	2	2	19	5.03	10.5
2015	0	10	10	38	10.07	26.3
2016	1	5	6	25	6.63	24.0
2017	0	11	11	40	10.58	27.5
2018	2	4	6	33	8.70	18.2
2019	2	4	6	25	6.57	24.0
2020	NA	3	3	21	5.51	14.3
2021	NA	7	7	33	8.63	21.2
2022	0	15	15	28	7.38	53.6
2023	0	10	10	33	8.33	30.3
2024	0	4	4	26	6.49	15.4
**Total**	**18**	**132**	**150**	**776**	**10.27**	**19.3**

NA: not applicable; TB: tuberculosis.

NA indicates years when the project was not conducted because of COVID-19 pandemic-related restrictions (2020–2021).

**Figure 2:**
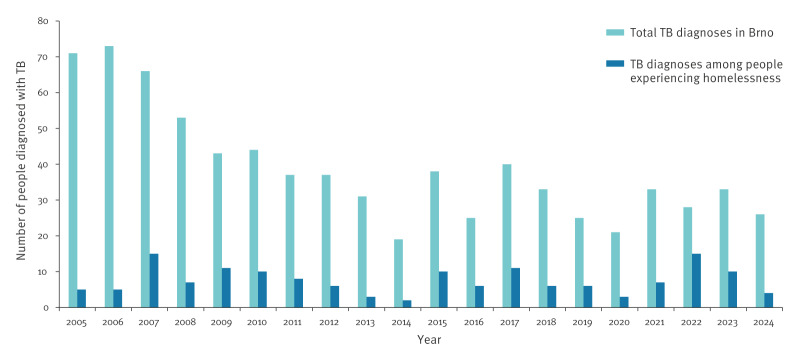
Annual number of people diagnosed with tuberculosis overall and among people experiencing homelessness, Brno, 2005–2024 (n = 776)

Figure 2 illustrates the long-term trend of declining TB incidence in the overall Brno population, contrasted with the persistent and often rising share of TB diagnoses among people experiencing homelessness. While overall TB numbers in Brno decreased over time, the relative burden in this vulnerable group remained consistently high.

Based on the total of 150 individuals experiencing homelessness who were diagnosed with TB between 2005 and 2024, and using the upper-bound estimate of 3,000 people experiencing homelessness in Brno, the average annual TB incidence in this population was ca 250 per 100,000. For comparison, the average TB incidence in the general Brno population during the same period was ca 10.3 per 100,000, based on annual surveillance data and population figures from the Czech Statistical Office [8]. This corresponds to a relative risk of 24.4 (95% CI: 20.5–28.9), meaning that people experiencing homelessness in Brno were over 24 times more likely to develop TB disease than the general population.

Finally, although they represent only 0.5–0.8% of Brno’s total population (based on estimates of the people who experience homelessness and the average total population between 2005 and 2024), individuals experiencing homelessness accounted for 19.3% of all reported TB diagnoses in the city during this period. 

## Discussion

Although only 18 individuals were diagnosed with TB through the Project, an additional 132 people experiencing homelessness were detected through other methods. In total, people without stable housing accounted for 19.3% of all TB diagnoses reported in Brno between 2005 and 2024, although they represented only 0.5–0.8% of the city’s population [8,9].

The results confirm that the risk of developing TB disease remains disproportionately high in this group. Based on surveillance data and population estimates, the average TB incidence among people experiencing homelessness was 24.4 times higher than in the general population (95% CI: 20.5–28.9), which aligns with other studies from Europe and elsewhere [4-6]. Structural and behavioural risk factors, such as delayed access to care, co-morbidities, substance use, poor nutrition, overcrowded shelters and stigma, probably contribute to this elevated risk [6,7]. Despite an overall decline in TB incidence in Brno and Czechia [1,14], the proportion of TB attributable to individuals experiencing homelessness increased, exceeding 50% in some years. This highlights the persistent vulnerability of this group and underscores the need for sustained outreach interventions even as national incidence rates decline.

A key strength of this project was its long-term implementation, which enabled longitudinal monitoring and intersectoral cooperation. Consistent partnership with the City of Brno and the involvement of municipal social workers helped reach individuals who would otherwise remain underserved. The use of stable screening methods and data collection tools allowed for comparability across time.

Notably, none of the 18 individuals diagnosed through the Project reported drug use or a history of incarceration – factors often associated with TB in populations of people who experience homelessness in western Europe [4-6]. This difference may reflect local epidemiological patterns and supports the need to adapt screening approaches to the social context.

The use of chest radiography proved essential, as all individuals diagnosed through the Project reported symptoms, but subclinical forms of TB may still have gone undetected without imaging. Subclinical TB, characterised by radiographic abnormalities in asymptomatic individuals, has been recognised as an important reservoir of transmission risk [2]. Our approach aligns with recommendations from WHO and the European Centre for Disease Prevention and Control (ECDC) to incorporate imaging in high-risk settings [15,16].

High-quality data were available for all confirmed TB diagnoses, thanks to mandatory national notification requirements [10,12]. These reports include demographic, clinical and behavioural information and allow for long-term follow-up. Although there was a limited level of missing data, all TB diagnoses were recorded using a consistent methodology, regardless of detection route. This strengthens the internal validity of the comparison.

Comparing the 18 individuals diagnosed through the Project with the 132 diagnosed through other means reveals important differences. While basic demographic profiles were similar, treatment outcomes differed. Among individuals identified through the Project, 13 completed treatment, two were lost to follow-up, and three died. In the group identified through passive or other methods, 80 completed treatment, 30 were lost to follow-up, and 22 died. In percentage terms, 72.2% of individuals identified through the Project successfully completed treatment, compared with 60.6% among those identified through other routes, and loss to follow-up occurred in 11.1% vs 22.7%, respectively. Even though this is based on low numbers, the suggest that earlier detection and close coordination between public health and clinical services may contribute to improved outcomes.

The Project's outreach and educational value should not be underestimated. Even in years without newly detected TB, participants received information about symptoms and access to care. These benefits are consistent with experiences from other European cities, where mobile outreach and repeated engagement have helped improve early diagnosis and reduce TB transmission [3,5,13].

However, some limitations must be acknowledged. Detailed demographic data were not collected for individuals who declined to participate. According to field staff and social workers, common reasons for non-participation included lack of interest, intoxication, psychiatric comorbidities and mistrust of authorities. This introduces a potential selection bias, possibly excluding the most marginalised individuals. This could have led to an underestimation of TB burden among the most vulnerable.

Another limitation concerns the follow-up and classification of individuals lost during treatment. Each person diagnosed with TB was monitored by the Regional Public Health Authority until treatment completion, death or loss to follow-up, typically over a 12-month period. Individuals who could not be traced were classified in the national TB register as ‘treatment discontinued – not traceable’, in accordance with Czech public health reporting protocols.

The study’s limitations further include reliance on programme-based data originally collected for administrative and public health purposes. Nonetheless, the findings reflect real-world conditions and the practical realities of TB control in vulnerable populations. Although our case definition included clinically diagnosed TB, reliance on chest X-ray and clinical judgement in some cases may still lead to under- or overestimation of true TB prevalence, particularly in the absence of bacteriological confirmation.

In recent years, the number of participants in the Project has declined, particularly following the COVID-19 pandemic. This reduction may have affected the number of people diagnosed with TB and illustrates the importance of maintaining outreach intensity. Furthermore, the Project was paused in 2020 and 2021 during pandemic-related restrictions, which is likely to have contributed to a temporary increase in passively detected cases – a trend seen elsewhere in Europe [1,14].

Despite year-to-year fluctuations, our findings confirm a consistently elevated TB incidence among people who experience homelessness across the 20-year period. This supports a sustained disproportionate burden in this group, even when considering variability in population estimates. The TB incidence in the general population of Brno helped contextualise these findings.

From a public health standpoint, the Project was implemented with modest resources. Diagnostic and treatment costs were covered by national health insurance, and the incentive system – meal vouchers – was funded by the City of Brno. Although no formal cost-effectiveness analysis was performed, our findings are in line with evidence from a recent systematic review, which concluded that active TB screening is likely to be cost-effective in populations with TB incidence exceeding 40 per 100,000, such as people experiencing homelessness [15]. The observed TB prevalence in the screened population (6.8/1,000) also exceeds the threshold of one per 1,000 suggested by WHO and ECDC for targeted screening in high-risk groups [16].

## Conclusion

This 20-year public health initiative demonstrates the feasibility, acceptability and long-term value of targeted TB screening among individuals experiencing homelessness in a low-incidence setting. The findings highlight that people without stable housing remain disproportionately affected by TB – accounting for nearly one-fifth of all TB diagnoses in Brno over two decades, despite representing less than 1% of the population. Although only 18 individuals were diagnosed with TB through the Project, all had active pulmonary disease and had not previously accessed medical care. Their early identification probably prevented further transmission. In addition to clinical detection, the screening served as an opportunity for outreach, health education and strengthening trust in public services – particularly important in a population that often faces barriers to care. The success of this long-standing intervention was made possible through close collaboration between public health authorities, the Brno City Municipality, healthcare providers, and social services. Continued intersectoral coordination, stable funding, and attention to social determinants of health will be critical to maintaining progress, reducing diagnostic delays and improving TB control in underserved urban populations.

## Data Availability

The data supporting the findings of this study are not publicly available due to privacy and legal restrictions related to personal health information. Access to anonymised data may be granted upon reasonable request to the corresponding author, subject to approval by relevant data protection authorities.
